# Hemochromatosis—How Not to Overlook and Properly Manage “Iron People”—A Review

**DOI:** 10.3390/jcm13133660

**Published:** 2024-06-23

**Authors:** Agnieszka Szczerbinska, Beata Kasztelan-Szczerbinska, Anna Rycyk-Bojarzynska, Janusz Kocki, Halina Cichoz-Lach

**Affiliations:** 1 Faculty of Medicine, Medical University of Warsaw, 61 Zwirki i Wigury Street, 02-091 Warsaw, Poland; 2 Department of Gastroenterology with Endoscopy Unit, Medical University of Lublin, 8 Jaczewski Street, 20-954 Lublin, Poland; 3 Department of Clinical Genetics, Medical University of Lublin, 11 Radziwillowska Street, 20-080 Lublin, Poland; janusz.kocki@umlub.pl

**Keywords:** hemochromatosis, iron overload, *HFE* gene mutation, non-*HFE* gene mutation, phlebotomy

## Abstract

Hemochromatosis (HC) is the main genetic disorder of iron overload and is regarded as metal-related human toxicosis. HC may result from *HFE* and rare non-*HFE* gene mutations, causing hepcidin deficiency or, sporadically, hepcidin resistance. This review focuses on *HFE*-related HC. The illness presents a strong biochemical penetrance, but its prevalence is low. Unfortunately, the majority of patients with HC remain undiagnosed at their disease-curable stage. The main aim of HC management is to prevent iron overload in its early phase and remove excess iron from the body by phlebotomy in its late stage. Raising global awareness of HC among health staff, teaching them how not to overlook early HC manifestations, and paying attention to careful patient monitoring remain critical management strategies for preventing treatment delays, upgrading its efficacy, and improving patient prognosis.

## 1. Introduction

Hemochromatosis (HC) (previous synonyms of the disease: hereditary, congenital, primary, idiopathic hemochromatosis) remains the most prevalent inherited metabolic disorder in humans. Its frequency is about 1 in 250 in Caucasians. It is the main genetic cause of iron overload and is regarded as metal-related human toxicosis [1]. The disorder was first described by Trousseau in 1865. Von Recklinghausen, who was a German pathologist, used the term hemochromatosis 24 years later [2,3]. In 1996, Feder et al. confirmed the location of the *HFE* gene on chromosome 6 [4]. Despite the growing research evidence, new guidelines, and comprehensive reviews recently released, clinicians still feel uncertain about the rules of the diagnostic approach and treatment of HC. Overdiagnosis and overtreatment in individuals who do not require iron removal remain a common problem in clinical practice. Therefore, our review is an attempt to provide medical practitioners with concise evidence-based recommendations for disease recognition and its accurate management. The present review concerns only *HFE*-related hemochromatosis and other forms of iron overload are not the subject of this work. It should be noted that the synonyms of HC mentioned above are no longer appropriate for defining the condition and should be avoided as a major cause of confusion. The term “hemochromatosis” itself implies an iron overload of genetic origin; therefore, qualifiers such as “hereditary”, “genetic” or “primary” are unnecessary. Since clarification of terms used in clinical practice is a key issue to start with in posing the appropriate disease diagnosis, new recommendations for HC classification developed by the International Society for the Study of Iron in Biology and Medicine (BIOIRON Society) should be widely adopted [5]. We intend to create an easily understood and unequivocal guide to help clinicians in proper HC management. 

### Body Iron Homeostasis and Risk Factors of Iron Overload

The *HFE* gene controls proper iron absorption in the gastrointestinal (GI) tract. Iron delivery to the blood is controlled by a peptide hormone hepcidin synthesized by hepatocytes and secreted into the blood plasma. A detailed description of cellular and tissue regulation of iron homeostasis is available elsewhere [6,7,8,9]. Briefly, hepcidin binding to ferroportin, the sole iron exporter in humans, blocks iron transport. In normal conditions, ferroportin exports iron from the duodenal enterocytes, hepatic and spleen macrophages, and iron-storing liver cells [10]. The hepcidin–ferroportin axis is engaged in systemic iron homeostasis by influencing hepatic hepcidin synthesis. Due to genetic mutations that cause hepcidin deficiency or interfere with its interaction with ferroportin, dietary iron absorption increases [11]. *HFE* gene mutations lead not only to enhanced iron uptake and its increased plasma concentrations, but also to progressive iron deposition in the cytoplasm of parenchymal cells of various organs and tissues (particularly hepatocytes, pancreatic cells, cardiomyocytes, skin, joints, and other organs), causing deleterious complications with multi-organ damage years later if left untreated [12,13]. The toxic impact of surplus iron stems from the development of oxidative stress with free oxygen radical generation (i.e., hydroxyl and peroxide radicals in the Fenton and Haber–Weiss reactions). They destroy intracellular membranes, trigger DNA breaks, and lead to cell injury. The usual body mechanisms of antioxidant defense which involve superoxide dismutase, catalase, and glutathione peroxidase become exhausted in the course of HC [14,15,16]. As a consequence, DNA damage occurs with the subsequent impairment of protein synthesis and deterioration in cellular integrity. 

Some factors may modify the natural course of the disease, its prevalence, and severity. They include individual patient features (age, gender, comorbidities) and environmental triggers (alcohol consumption, smoking, hepatotropic virus infections, hepatotoxic drugs, etc.) [17,18]. 

Even though the genetic defect is present at birth, the signs and symptoms of HC rarely occur until adulthood. Most individuals remain asymptomatic throughout their lives. Signs and symptoms of the disease in p.C282Y homozygotes usually occur over the age of 40 in men and the age of 50 in women [19]. The illness presents a strong biochemical penetrance, but its prevalence is low. Recent studies indicate that only 10–33% of p.C282Y homozygotes present with overt disease, less frequently in women (probably due to the physiological loss of iron during menstruation and pregnancy). Nevertheless, HC remains a relevant illness due to its high prevalence in the Caucasian population. Also, the results of a recent study confirmed that p.C282Y homozygotes suffer from more frequent complications and higher death rates regardless of phlebotomy treatment in comparison to patients without the *HFE* mutation [20]. 

Iron concentration is tightly regulated in the human body. As mentioned before, most of the iron absorption occurs in the duodenum and upper jejunum through mature enterocytes [21]. Once absorbed, iron must either be used or stored because humans do not have the physiological mechanisms to actively remove iron from the body. The only way to eliminate it is passive loss through menstrual blood, bleeding, exfoliation of epithelium, etc. 

Gender-related differences influence the penetration of HC. Men suffer from the disease two to three times more often than women, and they more often present serious complications of the disease including diabetes and liver cirrhosis. 

In summary, the main risk factors for overt HC include the following:

(a) *HFE* p.C282Y homozygosity—the greatest risk factor;
(b) positive family history for HC in the first-line relatives;
(c) Northern European ethnicity—the disease is less prevalent in populations of Afro-American, Hispanic, and Asian origin;
(d) male gender—men are susceptible to developing HC symptoms at an earlier age; however, females’ risk increases after menopause or a hysterectomy.

## 2. Gene Mutations in Hemochromatosis

Three main mutations of the *HFE* gene have been described. The most common of them is p.C282Y, when cysteine replaces tyrosine in position 282 of the *HFE* gene. This type of mutation occurs most often in the population of Northern Europe (1 in 220–250 individuals). The homozygosity of p.C282Y is observed in 0.3–0.6% of the European population and is responsible for iron overload in 90% of Caucasians [22,23]. The second mutation, which, in combination with p.C282Y, is responsible for another 5% of HC cases is p.H63D (substitution of histidine instead of aspartame). The global prevalence of p.C282Y is estimated at 1.9%; p.H63D at 8.1%; and compound heterozygosity p.C282Y/H63D at 1.97%. About 10% of the entire population are carriers of defective genes. The third *HFE* mutation is confirmed at nucleotide 193, where serine is replaced by cysteine at position 65 (p.S65C). Unlike p.C282Y, regardless of phlebotomy treatment, homozygosity for p.H63D or p.S65C usually leads to mild disease or no clinical consequences at all.

Mutations of other genes, the so-called non-*HFE*, are rare and occur mainly in inhabitants of the Mediterranean Sea area [24,25]. The forms of iron overload related to rare genetic mutations (non-*HFE*) are not the object of this work.

## 3. The Current Classification of Hemochromatosis

**Figure 1:**
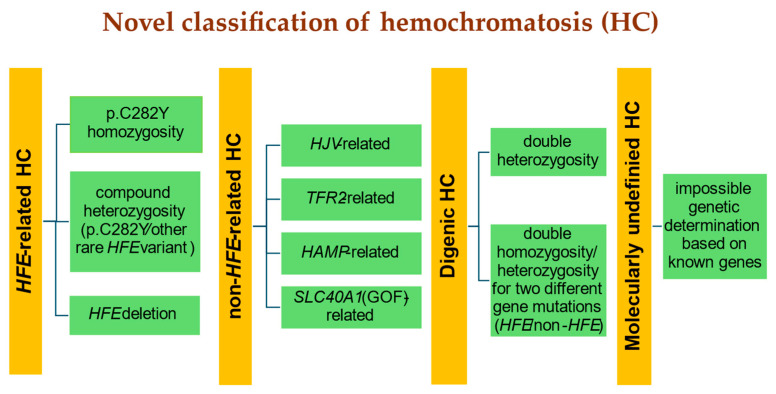
Novel classification of hemochromatosis (HC) proposed by the International Society for the Study of Iron in Biology and Medicine (BIOIRON Society); (adopted and modified from [5]).

Until recently, the HC classification was based on the criteria that included the type of genetic defect and the age at which the disease symptoms appear. In 2022, new recommendations for HC classification were developed by panelists of the International Society for the Study of Iron in Biology and Medicine (BIOIRON Society) during a meeting in Heidelberg, Germany. The novel classification presented in Figure 1 addresses both clinical and molecular issue involvement [5].

According to the novel classification, if a patient presents with convincing features of HC, but p.C282Y homozygosity (or compound p.C282Y/p.H63D heterozygosity) has not been revealed, a temporary “molecularly undefined” HC diagnosis should be established. Then, if indicated, treatment with phlebotomies may be initiated. The new HC classification takes into account the challenge of molecular clarification and facilitates medical communication.

## 4. Clinical Presentation of Hemochromatosis Regardless of Phlebotomy Treatment

Some individuals with HC never present the disease signs and symptoms. *HFE* homozygosity or compound heterozygosity does not ensure that signs and symptoms of iron overload will develop in an affected person during their lifetime. Furthermore, heterozygosity with a single *HFE* gene mutation does not create a risk of developing overt disease [26,27].

Women are more likely to develop HC symptoms after menopause when they no longer lose iron with menstruation and pregnancy. Since clinical signs of overt iron overload in homozygotes develop in adult life, testing in children is not recommended until the age of 18 [26,28].

Considering the variety of clinical symptoms, an HC diagnosis may be demanding, although a common initial presentation is a symptomless patient with mildly elevated liver enzymes [29]. Currently, overt HC is seldom seen in clinical practice due to routine assessment of iron indices. However, it may still cause a diagnostic challenge to non-expert doctors. Fatigue and arthralgias are currently the most common HC symptoms. The possible HC clinical manifestations are presented in Table 1. Nevertheless, most of them are uncommon due to early disease diagnosis and low clinical penetrance of HC.

Heterozygous *HFE* carriers (p.C282Y and p.H63D) usually do not manifest signs of the severe iron overload found in homozygous patients. Nevertheless, excessive iron accumulation and its related complications can develop in this subgroup of individuals. Anecdotal reports present cases of heterozygous HC and illustrate that they should be managed with caution, with close monitoring to prevent possible consequences of iron overload [30,31]. However, they should not be regarded as a general rule considering the redundant diagnostic investigations and overtreatment which still occur in homozygous p.H63D carriers. The majority of individuals require a genetic examination for the identification of heterozygous HC. The blood iron levels may remain normal due to the sufficient function of the healthy gene. Therefore, heterozygous carriers do not require treatment. Specific carriers of the heterozygous *HFE* variant may present additional causes for iron overload, but a simple heterozygous *HFE* mutation is not an indication of iron removal. Further studies are needed for a precise assessment of all the risk factors related to clinical penetration of the disease, its outcome, and the management requirements of heterozygous HC carriers. There is a strong indication for future observational and intervention studies to facilitate the early identification of subjects and their better management according to individual requirements. These are essential for future guidelines and suitable medical interventions in individuals with heterozygous *HFE* mutations.

### 4.1. Hemochromatosis and the Skeletomuscular System

Fatigue and joint pain occur as the most common early symptoms of the disease. Most HC patients develop a typical arthropathy, as described by Schumacher in 1964, in the metacarpophalangeal (MCP) and proximal interphalangeal (PIF) joints [32]. HC-related arthropathy can be observed in different joints, very often in the second or third MCP and the ankle joints, as well as the hip and knee [33]. Ankle arthropathy confirmed in 32–61% of HC patients can lead to foot pain during walking, swelling, and impairment of the range of motion (ROM) of the ankle [34]. It is often the first HC manifestation [35]. There are no particular clinical markers or radiological findings that allow reliable differentiation of HC-related arthropathy from primary ankle osteoarthritis. The diagnosis is mostly based on the patient’s history [34]. According to several reports, early initiation of iron depletion therapy might reduce the severity of HC-related arthropathy [36].

### 4.2. Hemochromatosis and the Central Nervous System

Rarely, patients with HC may present with a lack of energy (lethargy), irritability, memory fog, mood swings, depression, and anxiety. Moreover, due to iron accumulation in the brain, movement disorders (similar to Parkinson’s disease or chorea) and tremors (rarely) may occur [37,38]. Topiwala et al. found that alcohol consumption above 7 units per week led to higher iron content in the brain. Higher iron concentrations in the basal ganglia caused slower executive function and reaction time, and lower fluid intelligence. The authors indicated that iron accumulation in the central nervous system may constitute a potential reason for alcohol-induced cognitive impairment [39]. Therefore, abnormal iron homeostasis in patients with HC may be worsened by alcohol consumption, leading to overt motor and cognitive impairment.

### 4.3. Hemochromatosis and the Liver

Liver damage most frequently occurs in *HFE*-related hemochromatosis [22]. The clinical presentation is diverse and includes the following: asymptomatic increases in ALT and AST activity, followed by non-specific right upper quadrant epigastric pain, and finally, signs of end-stage liver disease. SF below 1000 ng/mL at the time of HC diagnosis identifies individuals who are at low risk of advanced liver fibrosis [26]. Several studies have confirmed the association between alcohol intake and liver cirrhosis development in HC patients. Alcohol downregulated the expression of hepcidin both in hepatoma cells in vitro, as well as in mice after short-term alcohol exposure [40]. Therefore, hepcidin-associated mechanisms involved in guarding against the detrimental impact of iron overload are disrupted by alcohol intake [41]. As reported elsewhere, both excess iron and alcohol consumption induce lipid peroxidation and oxidative stress. The liver is more susceptible to harm generated by oxidative stress than other organs [42]. Recent reports indicate that HC patients who consume above 60 g of alcohol per day have about nine times higher risk of liver cirrhosis development. Moreover, drinking above 80 g of alcohol per day significantly reduces patient survival [43,44]. However, the development of fulminant hepatic failure was also observed in a young patient with minimal alcohol consumption [45]. Risk factors linked to advanced liver disease in the course of HC include the presence of comorbidities (i.e., diabetes and arthropathy), alcohol misuse, high ferritin levels (above 1000 µg/L), increased liver iron concentration (LIC) (above 200 µmol/g), and phlebotomy (above 9.6 g of iron from stores) [26].

Patients with HC and liver cirrhosis also present an increased risk of hepatocellular carcinoma (HCC), which leads to about 45% of deaths in the aforementioned population. The relative risk of developing malignancy during HC ranges from 20 to 200%, and the risk factor is SF > 2000 ng/mL. The ten-year HCC incidence in HC patients with liver cirrhosis is 6–10%, and is higher in men than in women [46]. However, the most recent report by Schaefer et al. after an investigation of 8839 individuals from the Tyrol region in Austria revealed that HCC incidence was not significantly higher in p.C282Y homozygotes than in the controls matched for age and sex [47]. Recommendations for HCC screening in HC cirrhotics are the same as in cirrhotics of other etiology. An ultrasound hepatic evaluation with/without α-fetoprotein estimation every 6 months is required [48]. HCC is rather rare in non-cirrhotics with HC; however, patients should be closely monitored for signs suggestive of liver cancer even after the excess iron removal as the risk of HCC remains increased [49]. Therefore, the aforementioned reports support the implementation of cancer screening programs in the majority of patients with overt HC. Currently, based on international guidelines, the diagnosis of HCC (regardless of its etiology) relies on non-invasive criteria (multiphasic contrast-enhanced CT and MRI scanning) [48]. Notably, Bardou-Jacquet E. et al., in their retrospective study of patients with the p.C282Y mutation, discovered that advanced fibrosis of the liver can be reduced after treatment, and its regression to a stage F2 and below led to a significant decline in the long-term risk of liver cancer [50].

### 4.4. Hemochromatosis and the Cardiovascular System

Heart damage in the course of HC may manifest as cardiomyopathy (dilated or restrictive), arrhythmias (including sinus node dysfunction and atrial fibrillation), and heart failure. Heart hypertrophy or dilatation, systolic or diastolic dysfunction, and arrhythmias may occur as cardiac HC manifestations [51]. When symptoms of heart failure appear, the patient’s prognosis becomes poor. Cardiomyopathy is the second most common cause of death in patients with HC. Therefore, it is crucial to prevent heart complications in advance at the preclinical phase of the disease [52,53]. Cortés et al., in their retrospective study, observed the p.C282Y/p.H63D mutation’s coexistence with a higher frequency of heart infarction. However, the aforementioned mutation was related to a lower frequency of diastolic dysfunction in patients with a normal left ventricular ejection fraction (LVEF). No particular HFE mutation was reported to be related to LVEF below 55% [54]. Electrocardiography (ECG) is not very useful as a diagnostic tool since alterations occur in the late stage of cardiac HC. They are not specific and may include signs of left ventricle hypertrophy, low QRS voltage, nonspecific ST–T changes, and atrioventricular, supraventricular (mostly atrial fibrillation), or ventricular arrhythmias. The best non-invasive method for detecting cardiac iron deposits in a patient with HC is an MRI T2*. Iron acts as a paramagnetic force and disrupts the magnetic signals received by the detector, which is shown as an early darkening on MRI imaging [55]. Moreover, cardiac magnetic resonance (CMR) presents good sensitivity and specificity in assessing different types of cardiomyopathy. Therefore, it is used for the evaluation of cardiovascular morphology, chamber dimensions and volumes, myocardial perfusion, and heart function. However, if an MRI is not available, transthoracic echocardiography (TTE) can be a valid alternative for indirect evidence of heart involvement in HC. It is an inexpensive, non-invasive, and widely available method to screen patients for HC cardiac involvement. It is worth noting that TTE is not able to assess the cardiac iron collection. Phlebotomy exerts a beneficial impact on cardiac function, including left ventricular twist and torsion mechanics [51]. Emerging evidence indicates that ferroptosis, which is a non-apoptotic type of cell death, induces lipid peroxidation with impairment of membrane function and has a relevant influence on the development of HC-associated cardiomyopathy [56].

### 4.5. Hemochromatosis and the Endocrine System

Diabetes in HC develops as a result of pancreatic islet damage by iron overload as well as insulin resistance secondary to liver injury [57,58]. As reported, high iron content increases the risk of the development of type 2 diabetes mellitus (T2DM). It induces low insulin secretion, causes insulin resistance, and elevates hepatic gluconeogenesis [59]. However, based on the SF levels, we cannot foresee the risk of diabetes in p.C282Y homozygotes. The T2DM prevalence among p.C282Y homozygotes is relatively low based on the results of population screening programs [60,61,62,63]. It could be explained by the adoption of *HFE* genotyping after 1996 for early HC detection. As reported, phlebotomy treatment is not associated with T2DM improvement in HC [64].

Hypogonadism remains the second most prevalent endocrine problem in the course of HC. It is a consequence of selective iron deposition in the pituitary gland and causes alterations in hormonal secretion. It occurs most often in adolescent HC. Men may present with loss of libido, impotence, and osteoporosis, while women suffer from amenorrhea or premature menopause. Rare cases of hypopituitarism with adrenal, parathyroid, and thyroid gland alterations are also reported in the course of HC. Osteoporosis may occur in up to 25% of HC patients [65].

### 4.6. Hemochromatosis and the Skin

The disease has been called “bronze diabetes” due to changes in skin color and its coexistence with the disease of the pancreas. Hypermelanotic pigmentation is a typical sign of overt disease and is seen in more than 70% of patients. The altered skin color (bronze or gray) is primarily due to melanin rather than iron [66,67]. HC-related pigmentation occurs more often in sun-exposed and/or scarred skin areas. There are correlations between skin manifestations and other signs of the disease. Moreover, porphyria cutanea tarda (PCT) can be related to the excessive amount of hepatic iron with *HFE* gene mutation prevalence of 60–80%. Therefore, if a blistering condition occurs and porphyria is suspected, testing for HFE mutations should be performed to check for HC [68,69]. Also, pigmented purpuric dermatitis and alopecia areata (AA) affecting the beard have been described in a 56-year-old man with p.C282Y/p.H63D compound heterozygosity [70]. There are few reports of patients with HC and AA suggesting that AA may be an uncommon and early skin manifestation of HC, but their genetic interconnection is still unclear [71].

## 5. Diagnostic Approach to the Patient with Iron Overload Suspicion

**Figure 2:**
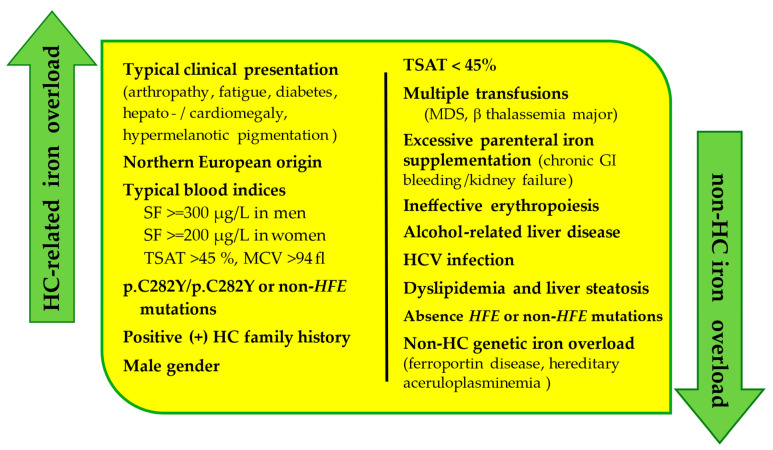
Differential diagnosis of iron overload (HCV—hepatitis C virus; HC—hereditary hemochromatosis; MCV—mean red cell volume; MDS—myelodysplastic syndrome; SF—serum ferritin; TSAT—transferrin saturation).

Timely management of individuals with suggestive laboratory and/or clinical presentation of iron overload enables early diagnosis, successful treatment, and prevention of serious complications. The diagnosis of HC is noninvasive and should include physical examination, evaluation of blood iron parameters, medical imaging, and genetic testing. Since iron overload may occur as a result of inherited or acquired factors, a differential diagnosis is important to distinguish it from other frequent causes of iron overload, such as metabolic dysfunction-associated steatotic liver disease (MASLD), formerly known as non-alcoholic fatty liver disease (NAFLD), alcohol-associated liver disease, or hepatitis C virus (HCV) infection [72]. Moreover, history of multiple transfusions (ß-thalassemia major, MDS); excessive parenteral iron supplementation (in chronic gastrointestinal bleeding or chronic kidney failure); ineffective erythropoiesis; as well as non-HC genetic iron overload (ferroportin disease, hereditary aceruloplasminemia) should also be excluded. The differential diagnosis in a patient with clinical presentation of iron overload is presented in Figure 2.

The diagnostic approach to a patient with HC suspicion may be challenging. Early disease symptoms such as stiff joints and fatigue are nonspecific for HC and may also be observed in other disorders. Since the only alteration seen in the majority of individuals with a *HFE* mutation is high blood iron levels, the disease may be recognized coincidentally based on incorrect results of blood tests performed for other indications or when screening family members of patients diagnosed with HC. As a rule, patients with a clinical presentation suggestive of HC and/or positive family should be referred for the assessment of serum iron parameters.

### 5.1. Blood Tests

The first step in the diagnostic approach for a patient with HC suspicion is the assessment of serum transferrin saturation and serum ferritin. The following are two crucial tests to detect iron overload:

1. Serum transferrin saturation (TSAT)—is calculated as the ratio between serum iron and total iron-binding capacity (TIBC). The higher-than-normal value of TSAT (above 45%) remains the earliest disease indicator present in all hemochromatosis subtypes [24]. However, increased TSAT is also observed in other disorders (e.g., hemolysis, cytolysis) or decreased blood transferrin concentration (e.g., hepatocellular failure, proteinuria, malnutrition, genetic alterations) [73]. Normal or even lower TSAT values can be observed in patients with ferroportin disease or hereditary aceruloplasminemia despite overt iron overload [74,75].
2. Serum ferritin level (SF)—is a commonly used diagnostic marker for the evaluation of iron storage in the body, although not very accurate [76]. The concentration of ferritin in the blood is influenced by many factors, as it is an acute-phase protein. Elevated SF levels (above 300 μg/L in men and postmenopausal women; above 200 μg/L in premenopausal women) require precise explanation before they are assigned to iron overload. Other conditions of hyperferritinemia, such as metabolic syndrome, alcoholism, inflammation, and marked cytolysis, should be ruled out [12]. Nevertheless, SF is an important prognostic factor in patients with HC. It is a predictor of advanced liver fibrosis and cirrhosis in patients with previously diagnosed hemochromatosis.

### 5.2. Genetic Testing

Based on the clinical picture or elevated levels of serum ferritin and transferrin, testing for *HFE* gene mutations remains a standard and confirmatory step of the diagnostic approach to individuals with HC suspicion. HC results from mutations causing hepcidin deficiency or hepcidin resistance. Both *HFE* and non-*HFE* mutations can induce hepcidin deficiency. *HFE*-related HC associated with hepcidin deficiency results from genetic p.C282Y homozygosity or p.C282Y/p.H63D heterozygosity (the so-called compound heterozygote). *HFE* p.C282Y homozygosity is found in more than 80% of cases of overt disease. Neither homozygous nor heterozygous p.H63D or p.S65C mutations lead to iron overload and are not relevant for the HC diagnosis [12,26,77]. As indicated, there is no consensus on the diagnostic value of p.H63D mutation. The EASL guidelines indicate that p.H63D is not a disease-inducing variant and individuals with this mutation can be managed based on their clinical signs and symptoms of iron overload, not their genotype alone. Also, environmental and other genetic background should be taken into account in this patient subgroup. However, according to the guidelines, in patients with p.C282Y/p.H63D heterozygosity or p.H63D homozygosity and confirmed by MRI R2/T2 or histopathology iron overload, phlebotomy is the treatment of choice even if results of randomized clinical trials are lacking [26,77].

Non-*HFE* HC related to hepcidin deficiency is rare and is related to genes encoding the proteins engaged in hepcidin synthesis, including the hemojuvelin (*HJV*), hepcidin (*HAMP*), and transferrin receptor 2 (*TFR2*) genes. Finally, a rare type of HC related to hepcidin resistance and caused by mutations in the ferroportin gene (*SLC40A1*) can alter the interaction between hepcidin and ferroportin [78]. Allele prevalence estimation is 74/100,000 for type 2A, 20/100,000 for type 2B, 30/100,000 for type 3, and 90/100,000 for type 4 hemochromatosis classified according to the previous criteria. Although less frequent, the non-*HFE* disease can be as serious a cause of iron overload as the *HFE* one [25]. Consequently, for individuals without p.C282Y mutation and no signs and symptoms of iron overload, the genes sequencing tests of the basic panel are recommended and should cover the following genes: rare *HFE* gene mutation, caeruloplasmin (*CP* gene), bone morphogenetic protein (*BMP6* gene), solute carrier family 40 member 1 (*SLC40A1* gene), the genes related to the early disease onset as *HAMP* gene and hemojuvelin (HJV gene) and those connected with abnormal transferrin, transferrin receptor 2 (*TFR2* gene) and transferrin (*TF* gene), and the hyperferritin-cataract syndrome gene (*FTL* gene). Since diagnosis based on gene panels is more easily available, the unbiased confirmation of mutation presence may help to avoid confusion and facilitate family screening [77]. Before genetic testing for non-*HFE* mutations, other causes of increased iron-related parameters should be excluded (i.e., alcoholic liver disease, MASLD, HCV infection), as they occur more often than the rare non-*HFE* HC [79,80].

### 5.3. Additional Diagnostic Assessment for Hemochromatosis

Additional diagnostic tests can be helpful to confirm the diagnosis and look for other HC-related problems:

1. Liver enzymes and function tests—the pattern of liver function alterations helps monitor liver damage in the course of HC.
2. Liver biopsy—determining the hepatic iron concentration (HIC) is rarely required to establish a final HC diagnosis; therefore, currently, genetic examination and imaging testing have replaced liver biopsy. Occasionally, it may be used to confirm or exclude other co-existing chronic liver diseases and to determine the degree of hepatic fibrosis, especially in cases with p.C282Y homozygosity and SF above 1000 ng/mL. HIC assessment may also be indicated in cases of suspected genetic iron overload with negative results towards common mutations including p.C282Y, p.H63D, and p.S65C. In remaining cases, liver biopsy is an option for individual consideration [81]. HIC assessed in a proper quality biopsy sample that is sample gross weight equal to or above 1 mg dry weight remains an accurate measure of entire hepatic iron concentration [82].
3. Magnetic resonance imaging (MRI)—the reference imaging technique for the evaluation and quantification of HIC; a noninvasive and accurate alternative to a liver biopsy with an excellent correlation between both aforementioned procedures; useful for identification of iron overload, its rating, and the assessment of treatment results [83,84].
4. Superconducting quantum interference device biomagnetic liver susceptometry (SQUID-BLS)—a noninvasive diagnostic device with very limited availability, used for HIC assessment; since hepatic iron can change its magnetic susceptibility, SQUID-BLS can quantify liver magnetic susceptibility and therefore determine liver iron concentration [80,85]. The procedure is available in very few specialized centers only.

## 6. Screening of Healthy People for Hemochromatosis

Screening for *HFE* mutations is not recommended in the general population. However, genetic evaluation may be required in patients with chronic liver disease with unclear etiology and TSAT above 45%. Moreover, it can be considered in individuals with type 1 diabetes mellitus, HCC, porphyria cutanea tarda, or chondrocalcinosis [23,86]. There is a recommendation to test first-degree relatives of HC patients with *HFE* mutation, not only due to potential genetic complications but also similar environments that may influence the mutation penetrance. For children of one HC parent confirmed with *HFE*-related HC, testing for *HFE* gene mutations in the other parent is recommended. If it is not detected, the child is heterozygous and does not need to be tested. If testing of the other parent is not possible, the child does not need to be diagnosed before the age of 18 because clinical symptoms of HC rarely occur in childhood. Furthermore, there is no need to check for *HFE* gene mutations in the pediatric population before maturity if they have first-degree relatives with HC [28].

Results of a recent cross-sectional study conducted on 86,601 participants revealed the possibility of genomic screening to recognize latent iron overload and promote appropriate management. They also confirmed the advantage of population screening for *HFE* p.C282Y homozygosity, which was uncovered in 201 participants. Up to 57 of them (28.4%) were previously diagnosed with HC type 1, and screening of the remaining 144 individuals revealed the presence of mutation [87]. However, additional studies are required for the assessment of health outcomes and the cost-effectiveness of genomic screening. 

## 7. Treatment of Hemochromatosis

Treatment of HC is focused on iron overload prevention in the early-phase disease and the elimination of surplus iron from the body by phlebotomy in its late stages. Since there are no specific pathways of iron elimination in humans, excess iron may be successfully removed by multiple bloodlettings. Therefore, the treatment of choice in HC patients is therapeutic phlebotomy [26]. Homozygotes/compound heterozygotes should be assessed annually, and potential iron overload symptoms should be checked. They may include weakness, arthralgia, liver dysfunction, heart failure, arrhythmia, or testicular atrophy.

### 7.1. Phlebotomy—The First-Line Treatment for Iron Depletion

Therapeutic phlebotomy (venesection or ‘bloodletting’) remains the recommended first-line treatment of choice for HC [88]. As reported elsewhere, the disease can be treated safely and effectively by removing iron from the body by repeated phlebotomies ordered based on the patient’s age, overall health, and the grade of iron overload. Treatment should be initiated when the presence of iron overload is confirmed. Its main indicators include serum iron parameters such as TSAT above 45% and ferritin above 200 μg/L in females and TSAT above 50% and ferritin above 300 μg/L in males and postmenopausal women [26].

Initial treatment may require about 500 mL of blood removal (each containing 240–250 mg iron) every 1 to 2 weeks. Before each blood removal, the hemoglobin level should be checked, and its level should not drop below 11 g/dL. If its value is below 11 g/dL, the procedure should temporarily be stopped; if it is 11–12 g/dL, the frequency should be reduced. The ferritin concentration should be determined after four phlebotomies until it reaches 200 µg/L, then every one to two phlebotomies until it decreases to 50 µg/L [89]. The next step is maintenance treatment. Once the body iron content decreases, blood can be removed less frequently, generally two to four times a year, and it should follow the patient’s need. Since elevated TSAT levels can be observed despite iron reduction, high TSAT is not regarded as a treatment target in patients with *HFE*-related HC. According to the EASL guidelines, ferritin levels below 50 μg/L in the induction phase and below 100 μg/L in the maintenance phase of treatment remain targets for phlebotomy [26]. Before blood removal, the patient should be well hydrated and avoid excessive physical activity for 24 h after the procedure. As reported, adverse effects of phlebotomy could occur in about 50% of patients including phlebitis, malaise, hematoma, delayed bleeding, infection, neurovascular damage, and fatigue [29].

Some patients maintain normal iron levels while others may require blood removals at regular monthly intervals. The schedule of phlebotomies should be tailored individually according to the rate of iron accumulation in the body.

The treatment can help relieve patient signs and symptoms and prevent multi-organ complications observed predominantly in the liver, pancreas, and heart. Phlebotomy may delay the disease progression and, in some cases, even reverse it.

### 7.2. Erythrocytapheresis as an Alternative to Phlebotomy

Erythrocytapheresis (EA) is an extracorporeal blood separation procedure by which erythrocytes (RBC) are selectively removed, and the remaining blood is returned to circulation. It aims to reduce RBC count and/or iron content [90]. In comparison to phlebotomy, EA removes red blood cells more efficiently and diminishes iron overload more rapidly. Therefore, it is cost-effective, particularly in the induction HC treatment—fewer procedures are needed in longer treatment intervals. Personalized EA may represent the preferred therapeutic option, if available, for suitable and selected HC patients presenting good antecubital venous access and fair cardiac function [88,91,92]. EA is suggested to be more suitable for subgroups of HC patients with cardiac disease as it is related to insignificant hemodynamic alterations in comparison with phlebotomy, but also to individuals with low platelet count or low protein levels as only RBCs are removed during EA and other blood constituents return to the body [77].

### 7.3. Iron Chelators as a Therapeutic Alternative in Hemochromatosis

Iron chelation therapy is recommended for selected patients with HC. Patients with severe iron overload and no proper response to phlebotomy treatment, severe cases of non-*HFE* HC (juvenile type), presenting with poor vein conditions and/or, anemia, or heart complications may have contraindications to venesection therapy. Instead, iron chelators may be administered, although they are not routine indications in hemochromatosis [77,93].

Administration of chelating agents can be oral, subcutaneous, or intravenous, favoring the elimination of toxic iron in the urine or the feces through the bile route. In Europe, three iron chelators are available, but availability varies between countries [94]. Iron chelators for clinical use include deferoxamine (DFO), deferiprone (DFP), and deferasirox (DFX). All three aforementioned medications have been approved by the United States Food and Drug Administration. Each of them has its advantages and disadvantages [95]. The DFO, a nontoxic parenteral iron chelator is accepted for long-term treatment in clinical settings. The DFP as an oral medication remains a good alternative for cases with no response to DFO or DFX. The DFX, an oral tridentate iron-chelating agent, binds iron in a 2:1 ratio [96,97]. Some recent reports suggest that the administration of deferasirox is more affordable than conventional deferoxamine therapy [98]. In general, deferasirox remains the first choice, and deferiprone and deferoxamine are considered the second and third therapeutic options, respectively [99].

### 7.4. Proton Pump Inhibitors—An Adjunct Therapy for Hemochromatosis?

Non-heme iron absorption can be lowered by proton pump inhibitor (PPI) treatment, therefore it may be considered an HC adjunct therapy. Results of the recent randomized controlled trial revealed significantly reduced indications for phlebotomy in patients with p.C282Y homozygosity during 12 months of pantoprazole treatment (40 mg/day) in comparison with the controls (placebo). Concerning the PPIs’ long-term safety, they might perfectly complement conventional HC therapy in cases where medical indications for PPIs are present [100]. The aforementioned results were confirmed by a meta-analysis that involved 68 HC patients (34 in the PPI group and 34 in the placebo or non-PPI group). The minimal time of PPI treatment was the same (1 year) for all the patients [101]. However, regular PPI treatment is not currently recommended in patients with HC.

### 7.5. Liver Transplantation for Hemochromatosis

Liver transplantation (LT) is used for HC patients with advanced liver failure or HCC development. Nevertheless, HC remains an infrequent indication for LT and concerns about 1% of all transplants [27]. This kind of treatment normalizes iron metabolism due to the proper hepcidin synthesis in the donor’s liver. Recently, the analysis of data from the United Network for Organ Sharing registry on survival after LT between 2003 and 2019 in adults with HC was published. It revealed that the post-LT survival rates for HC were outstanding and similar to the other liver transplant recipients [102]. Moreover, patients with HC complicated by HCC development might reach survival rates analogous to other liver diseases following curative LT. The Barcelona Clinic Liver Cancer (BCLC) Staging System allows the precise evaluation of primary liver cancer, and well-selected patients present very good five-year post-LT survival [103].

### 7.6. Recommended Lifestyle and Diet Modifications in Patients with Hemochromatosis

Patients who suffer from HC should be educated on how they may reduce the risk of the disease progression, but they should also know and remember that the disease cannot be treated simply by diet. Dietary restrictions should not substitute for iron removal treatment. The recommendations regarding their lifestyle and diet include the following precautions:

- withdrawal of additional iron sources such as iron supplements, iron-containing multivitamins, and iron-fortified foods and drinks (e.g., breakfast cereals, sports energy bars, etc.);
- recommendation of a varied vegetarian, semi-vegetarian, or flexitarian diet;
- avoidance of vitamin C supplements which increase iron absorption, but there is no need to restrict natural vitamin C in their diet (fruit and vegetables); fruit juices should be consumed between meals;
- recommendation of complete alcohol abstinence as its hepatotoxic impact aggravates liver damage; there is no safe alcohol amount;
- elimination of raw or undercooked fish and shellfish from the diet due to the risk of infections (hepatitis A and E virus, Norovirus, *Listeria monocytogenes*, *Campylobacter*, *Salmonella*, and *Escherichia coli*) [104,105,106,107].

Any additional extreme modifications of diet are not usually required for HC patients who receive blood removal treatment. Multicenter, prospective, randomized studies should further clarify the impact of dietary interventions in patients with HC [108].

When iron overload has been diagnosed, patients are recommended for lifelong monitoring of their iron-associated indicators.

## 8. Hemochromatosis in Women—Pregnancy and Fertility Issues

Although HC is not a gender-specific disease, it affects more men than women. Overt clinical signs and symptoms of the disease occur later in women in comparison with men [109,110].

Therefore, most women may not be aware of the disease until their middle age, often after already passing childbearing years. However, some of them may have a relative with HC and discover their disorder earlier before any signs and symptoms appear. The diagnosis of HC may also be established incidentally while they are examined due to other medical conditions or as part of a routine blood examination. Rarely, women of all ages may develop marks of iron overload, and their condition can be detected and confirmed at that time.

Results of a recent retrospective study on the HC impact on maternal and perinatal outcomes have been published. Data from over 36 million delivery hospitalizations revealed that there was a significant increase in HC prevalence over the studied period. A higher prevalence of gestational hypertensive complications and venous thromboembolism were observed in pregnant women with HC. Moreover, patients with HC had a longer hospital stay and higher total charges [111].

As suggested, pregnancy might temporarily reduce female iron concentration (sometimes with anemia development). Nevertheless, the described effect does not seem to lead to any long-term advantage for female iron control. On the other hand, Scotet et al. reported that pregnancy does not influence iron parameters and does not protect against ongoing iron-gathering [112]. Results obtained from the *HFE*−/− mice animal model also indicate that multiple pregnancies do not decrease body iron content [113]. These results correspond to other experimental observations and may suggest the possible impact of distinct factors like hormones on different disease presentations in men compared with women [114,115,116].

Pregnant women presenting with symptoms of mild or moderate iron overload without advanced liver dysfunction should be carefully assessed for individual therapeutic decisions, but for most of them, phlebotomy can be discontinued during the whole period of pregnancy to avoid sideropenia. One should keep in mind that even women with HC may develop sideropenia during pregnancy. As a rule, iron supplements should be avoided in HC; however, if iron deficiency occurs, pregnant HC patients should be managed similarly to other pregnant women.

In the past, pregnant women with HC were thought to have no higher risk of complications in comparison to the general population. Nevertheless, accumulating evidence indicates that feto-maternal complications may occur as a consequence of iron overload. A recent systematic review revealed that the risk of feto-maternal complications was higher even in the absence of altered iron parameters, therefore prenatal monitoring was strongly recommended [117]. As reported, the absorption of lead and iron increased the risk of preeclampsia, gestational hypertension, fetal congenital abnormalities, and growth problems. There is a higher risk of fetal neurodevelopmental delays and childhood leukemia in the course of HC. Therefore, close monitoring of both mother and baby is required in every case with HC mutation.

More research exploring the impact of HC on pregnancy and fertility is required.

## 9. Future Directions in Hemochromatosis

Few studies are being conducted on HC at present. Only two currently open and recruiting trials are registered on the website, https://clinicaltrials.gov/ (accessed on 14 May 2024). The first one is an interventional study held in France and focused on the selection of the best criteria in guiding bloodletting according to the current guidelines based on serum ferritin levels in comparison to both transferrin saturation and serum ferritin levels (NCT04779593). The second one, which is an observational study, aims to determine the prevalence of endocrinopathies in patients with HC and iron overload (NCT06137079). Results of further research are eagerly awaited to provide evidence that would warrant the prediction of the individual risk of iron overload and could enable physicians to initiate personalized management of patients with *HFE*-related HC.

## 10. Conclusions

Untreated HC continues to be a life-threatening disorder that may lead to increased morbidity and mortality due to iron overload. Unfortunately, the majority of patients with HC remain undiagnosed at the disease-curable stage. Undiagnosed, progressive iron accumulation may finally result in liver, heart, and endocrine failure. Therefore, early identification of patients with clinical features suggestive of HC and their appropriate management remains fundamental to preventing multi-organ damage with further clinical complications and ensuring normal life expectancy. This goal could be achieved by targeted case detection, although previous population screening studies have revealed that HC penetrance is lower than expected [118]. However, screening among at least Caucasian men of Northern European ancestry seems to be reasonable. Raising global awareness of HC among health staff, teaching them how not to overlook early HC manifestations, and creating well-organized patient surveillance remain crucial for preventing treatment delays, upgrading its efficacy, and improving patient prognosis.

## Figures and Tables

**Table 1:** Possible clinical presentation of hemochromatosis.

Organ/System	Symptoms
Skeletomuscular system	arthralgia, arthritis, chondrocalcinosis, reduced bone mineral density, fatigue, weakness
Central nervous system	lack of energy (lethargy), irritability, memory fog, mood swings, depression, anxiety, movement disorders, tremors
Liver	high liver enzymes, hepatosplenomegaly, liver fibrosis and cirrhosis, hepatocellular carcinoma
Cardiovascular system	cardiomyopathy, arrhythmia, heart failure
Endocrine system	hypogonadism, testicular atrophy, reproductive disorders with loss of libido, impotence, amenorrhea, hyperglycemia, diabetes mellitus, hypopituitarism
Skin	bronze or gray skin tone (hypermelanotic pigmentation), hair loss, porphyria cutanea tarda (?)
Immune system	immune defects, infections

## Data Availability

All data are included in the main text.
